# Analysis of mortality in François’ langurs (*Trachypithecus francoisi*) managed care in *Trachypithecus francoisi* rare animal breeding Center in Wuzhou, Guangxi, China: a 16-year review

**DOI:** 10.3389/fvets.2024.1376265

**Published:** 2024-08-14

**Authors:** Yi Xiong, Guanping Xie, Yifeng Li, Yasheng Mo, Zhengjun Wu, Youbang Li

**Affiliations:** ^1^Key Laboratory of Ecology of Rare and Endangered Species and Environmental Protection, Guangxi Normal University, Ministry of Education, Guilin, China; ^2^Guangxi Key Laboratory of Rare and Endangered Animal Ecology, College of Life Science, Guangxi Normal University, Guilin, China; ^3^Research Institute of Garden Plants and Animals, Wuzhou, China; ^4^Taihe Park, Wuzhou, China

**Keywords:** primates, François’ langur, managed care, mortality review, pathological

## Abstract

In managed care settings, primates are susceptible to a variety of health complications. A comprehensive understanding of the causes of mortality and their association with management practices is essential for enhancing the welfare of managed care populations such as François’ langurs (*Trachypithecus francoisi*). However, literature addressing prevalent diseases or causes of death in such settings remains limited among François’ langurs in managed care. To address this knowledge gap, we conducted an analysis of mortality causes in François’ langurs (*n* = 97) who died of natural causes during a 16-year period (2007–2022) at the *Trachypithecus francoisi* Rare Animal Breeding Center in Wuzhou, Guangxi, China. Morphological diagnosis and organ system and etiological evaluations were performed. François’ langurs were divided into six age-range groups, following previous studies: infant (≤ 1 year old), juvenile (1 to 2 years), sub-adult (2 to 4 years), adult (4 to 10 years), middle-aged (10 to 15 years), and geriatric (> 15 years). Results revealed that the primary causes of mortality in managed care François’ langurs were pneumonia (*n* = 11, 12.22%), neoplasia (*n* = 7, 7.78%), ileus (*n* = 7, 7.78%), senility (*n* = 6, 6.67%), gastroenteritis (*n* = 6, 6.67%), cardiac disease (*n* = 5, 5.56%), hemorrhage (*n* = 5, 5.56%), intestinal adhesion (*n* = 4, 4.44%), and renal abscess (*n* = 4, 4.44%). The gastrointestinal system was most frequently implicated in deaths, followed by the respiratory system (*n* = 17, 18.89%), multisystem disease (*n* = 16, 17.78%), and cardiovascular system (*n* = 15, 16.67%). Regarding etiology, infectious or inflammatory (*n* = 32, 35.56%) and physiological factors (*n* = 17, 18.89%) were identified as the leading contributors to the high mortality rate. It is imperative for managers to recognize the distinct risk profiles associated with different age groups. Specifically, pneumonia was the principal cause of death in infant and juvenile langurs, while renal disease, neoplasia, gastroenteritis, and intestinal obstruction were the primary causes of death in adult and middle-aged François’ langurs and advanced age and cardiac disease were the main causes of death in geriatric langurs.

## 1 Introduction

The François’ langur (*Trachypithecus francoisi*) is a highly endangered primate and is currently listed in the IUCN Red List of endangered species and as a priority protected animal in China (1). This unique primate is native to both tropical and subtropical limestone areas, as well as river valley karst cliffs. It is the most northerly distributed Asian species of langur monkey, with primary habitats located in Guangxi, Chongqing, Guizhou, and northern Vietnam (2). Compared to other primates, François’ langurs are characterized by a narrow distribution range and distinct habitat preferences. Its global population has experienced a sharp decline, raising the possibility of extinction. This decline is primarily due to habitat loss and fragmentation, exacerbated by human activities and hunting. Efforts to conserve the François’ langur have centered around the establishment of national wildlife reserves and dedicated breeding programs. Despite these measures, the species remains one of the more challenging primates to breed in managed care (3). The langurs, well-adapted to life in mountainous and forested areas, exhibit vigorous activity and specific dietary preferences, such as a variety of leaves and wild fruits. These characteristics necessitate stringent conditions for artificial managed and pose considerable challenges in replenishing populations within their ideal habitats. The practice of artificially breeding François’ langurs, while long-established, initially relied on conventional breeding methods due to limited understanding of their ecology, behavior, and habits, resulting in suboptimal survival rates. However, after more than four decades of dedicated *ex-situ* conservation, the François’ langurs population has experienced growth. Nevertheless, this success comes with its own challenges, notably the increasing prevalence of diseases that affect these managed care populations of François’ langurs.

Given the increasing incidence of disease in managed care populations of François’ langurs, it is imperative for veterinarians to deepen their understanding of the natural pathology of this species to provide high-quality treatment and care. Mortality data for managed François’ langurs remains limited. An individual case reports and brief case series, although gastrointestinal disorders (4, 5), nephrotic syndrome (6, 7), mycoplasmal and bacterial mixed infections (8–10), malignant neoplasia (11–16), and hepatapostema (17) have been identified in previous publications. Health threats to managed François’ langurs are a concern not only for the affected individual and zoological collection but also for the broader objectives of reproduction and conservation of the species.

To fully explore the causes of morbidity among managed François’ langurs (*n* = 97) held at the Institute of Garden Fauna and Flora in Wuzhou City, Guangxi Province, China, from 2007 to 2022, focused on disease type, age of onset, sex, and disease body. This study should provide a scientific basis for disease prevention and population management of François’ langurs in artificial breeding environments.

## 2 Materials and methods

### 2.1 Animals

This study examined a total of 97 François’ langur fatalities recorded over a 16-year period (2007–2022). The data were derived from necropsy reports from the Research Institute of Garden Plants and Animals (*Trachypithecus francoisi* Rare Animal Breeding Center) in Wuzhou, Guangxi, China. This institute started raising François’ langurs in March 1973 and was officially designated as a rare animal breeding center for François’ langurs in 1991 (18). According to the meteorological department, Wuzhou City, characterized by a subtropical climate, experiences year-round high temperatures, prolonged sunshine, substantial humidity, and abundant rainfall, closely resembling the environmental conditions favorable to François’ langurs, it has ideal climatic conditions and rich feed sources (19). François’ langurs were housed in open-top corrals made of wire and concrete, offering separate spaces for indoor and outdoor activities. Each room of enclosure featured a 1.3-m-high resting stand and an electric heater for warmth during colder periods. The outdoor area included three 1.5-m-high resting platforms and various climbing structures such as swings, wooden houses, and iron ropes for play and activities. The open-air activity field was enclosed by cement walls on both sides, with the top and front secured with barbed wire, facilitating observation by the keepers. A water pipe, providing flowing water continuously, was situated in front of the housing area. The feeding schedule included two daily meals (11:00 and 16:00) consisting of browse, green fodder, vegetables, and fruits. Additionally, as part of their enrichment, the monkeys were also given extra peanuts, red dates, and other grains.

### 2.2 Records review

Pathological reports of François’ langur deaths (*n* = 97), provided by the *Trachypithecus francoisi* Rare Animal Breeding Center of Wuzhou, Guangxi, China were reviewed. Each case was categorized according to information from the pathological report, including the studbook number, cause of death location and year of death, sex, age and pathology. Some death reports were limited, containing only partial pathological reports, medical records, presumed causes of death, or incomplete gross pathology or histopathology. Cases with ambiguous causes of death or lacking critical information were excluded from further statistical analysis.

The DAMNITV classification scheme used by Rizzo et al. (20) and Laurence et al. (21) was applied to adjust the etiological categories to determine cause of death. The body system categories were modified from Robinson et al. (22). While secondary and sporadic pathological findings were noted, only the primary significant etiological factors were assigned to each case based on several criteria: (i) Deaths resulting from traumatic bleeding, regardless of the organ involved, were categorized under the cardiovascular system; (ii) instances of multiple organ failure or lesions caused by aging and physical decline were classified as senescence; and (iii) diseases affecting multiple organs or the whole body, such as senescence, malnutrition, multiple organ abscesses, and hyperthermia, were classified as multiple systems.

### 2.3 Data analysis

Data were entered into spreadsheets categorized by organ system, cause of death, and morphological diagnosis. Every analysis was carried out with SPSS.26. Fisher’s exact tests for independence were applied to assess the significance of sex, age, and season in etiology. A 95% confidence interval was established and *p*-values less than 0.05, adjusted with Bonferroni correction, were considered significant. However, due to insufficient sample size in various categories, instances where Fisher’s exact test failed to yield *p* < 0.05, particularly when the count of affected animals was arbitrarily set to either female or male, were not included in the analysis. For further analysis, the top nine morphological diagnoses were designated as ‘Morphologic Diagnostic Type’ variables. Age groups were categorized as six age-range groups, following previous studies (23, 24): infant (≤ 1 year old), juvenile (1 to 2 years), sub-adult (2 to 4 years), adult (4 to 10 years), middle-aged (10 to 15 years), and geriatric (> 15 years), while seasons were divided into spring, summer, autumn, and winter. Age-season variables, combining age and season, were used alongside morphological diagnostic type variables to form a matrix for corresponding analysis. Data were then processed in SPSS.26, followed by Chi-square tests (test level α = 0.05) and cumulative inertia measurements. A cumulative inertia ratio greater than 0.5 indicated a favorable dimensionality reduction effect (25). This process yielded a summary, row point overview, column point overview, and corresponding analysis diagrams.

## 3 Results

### 3.1 Study population

The deceased François’ langurs (*F* = 46, *M* = 49, unknown = 2) from 2007 to 2022 were evaluated. Cases with unknown sex, age, and etiology were removed from statistical analysis, resulting in a final analytical sample size of 90 (*F* = 42, *M* = 48). The age groups were as follows: infant (*n* = 13; *F* = 4, *M* = 9), juvenile (*n* = 2; *F* = 1, *M* = 1), sub-adult (*n* = 6; *F* = 1, *M* = 5), adult (*n* = 29; *F* = 12, *M* = 17), middle-age (*n* = 16; *F* = 10, *M* = 6), and geriatric (*n* = 24; *F* = 14, *M* = 10). The number of animals by age-group and sex are summarized (Table 1).

**Table 1:** Total number of François’ langurs by age-group and sex (undetermined causes not included; *n* = 90).

Age group	Total	Female	Male
Infant	13	4	9
Juvenile	2	1	1
Sub-adult	6	1	5
Adult	28	11	17
Middle-age	16	10	6
Geriatric	25	15	10
Total	90	42	48

### 3.2 Morphological diagnoses

Total population mortality was categorized by morphological diagnosis, affected systems and organs, and etiology. The frequency of morphological diagnoses causing death (in total numbers and percentages), as well as the count and percentage of males and females and their distributions across age groups is outlined (Table 2). The nine most common diagnoses accounted for 58.89% of all deaths, and included pneumonia (*n* = 11, 12.22%), neoplasia (*n* = 7, 7.78%), ileus (*n* = 7, 7.78%), senescence (*n* = 6, 6.67%), gastroenteritis (*n* = 6, 6.67%), cardiac disease (*n* = 5, 5.56%), hemorrhage (*n* = 5, 5.56%), intestinal adhesion (*n* = 4, 4.44%), and pyelonephritis (*n* = 4, 4.44%).

**Table 2:** Morphologic diagnoses in all François’ langurs by sex and age group in descending order of occurrence (undetermined causes not included; *n* = 90).

Morphological diagnosis	Total		Sex		Infant		Juvenile		Sub-adult		Adult		Middle-age		Geriatric	
	*n*	%	F	M	*n*	%	*n*	%	*n*	%	*n*	%	*n*	%	*n*	%
Pneumonia	11	12.22	2	9	7	63.64	1	9.09	0	N/A	1	9.09	0	N/A	2	18.18
Neoplasia	7	7.78	4	3	0	N/A	0	N/A	0	N/A	4	57.14	1	14.29	2	28.57
Ileus	7	7.78	3	4	0	N/A	0	N/A	0	N/A	3	42.86	2	28.57	2	28.57
Senescence	6	6.67	5	1	0	N/A	0	N/A	0	N/A	0	N/A	0	N/A	6	100.00
Gastroenteritis	6	6.67	3	3	1	16.67	1	16.67	0	N/A	2	33.33	2	33.33	0	N/A
Cardiac disease	5	5.56	0	5	0	N/A	0	N/A	0	N/A	1	20.00	1	20.00	3	60.00
Hemorrhage	5	5.56	4	1	0	N/A	0	N/A	1	20.00	1	20.00	1	20.00	2	40.00
Intestinal adhesion	4	4.44	1	3	0	N/A	0	N/A	2	50.00	1	25.00	0	N/A	1	25.00
Pyelonephritis	4	4.44	4	0	0	N/A	0	N/A	0	N/A	1	25.00	3	75.00	0	N/A
Parasitosis	3	3.33	2	2	0	N/A	0	N/A	0	N/A	2	66.67	1	33.33	1	33.33
Septicemia	3	3.33	1	2	0	N/A	0	N/A	0	N/A	2	66.67	0	N/A	1	33.33
Hepatapostema	3	3.33	2	1	0	N/A	0	N/A	1	33.33	2	66.67	0	N/A	0	N/A
Conjunctivitis	2	2.22	1	1	0	N/A	0	N/A	0	N/A	1	50.00	1	50.00	0	N/A
Multiple organ abscesses	2	2.22	0	2	0	N/A	0	N/A	1	50.00	0	N/A	0	N/A	1	50.00
Hyperthermia	2	2.22	1	1	2	100.00	0	N/A	0	N/A	0	N/A	0	N/A	0	N/A
Myocarditis	2	2.22	0	2	0	N/A	0	N/A	0	N/A	1	50.00	1	50.00	0	N/A
Inanition	2	2.22	2	1	2	100.00	0	N/A	0	N/A	0	N/A	0	N/A	0	N/A
Nephritis	2	2.22	2	0	0	N/A	0	N/A	0	N/A	1	50.00	0	N/A	1	50.00
Foreign body asphyxia	2	2.22	0	2	0	N/A	0	N/A	1	50.00	0	N/A	0	N/A	1	50.00
Intestinal perforation	1	1.11	0	1	0	N/A	0	N/A	0	N/A	1	100.00	0	N/A	0	N/A
Intussusception	1	1.11	0	1	0	N/A	0	N/A	0	N/A	1	100.00	0	N/A	0	N/A
Hernia	1	1.11	1	0	0	N/A	0	N/A	0	N/A	1	100.00	0	N/A	0	N/A
Gastroatonia	1	1.11	0	1	1	100.00	0	0.00	0	0.00	0	0.00	0	0.00	0	0.00
Gastrectasis	1	1.11	0	1	0	0.00	0	0.00	0	0.00	1	100.00	0	0.00	0	0.00
Pancreatitis	1	1.11	1	0	0	0.00	0	0.00	0	0.00	0	0.00	1	100.00	0	0.00
Hepatic cirrhosis	1	1.11	0	1	0	0.00	0	0.00	0	0.00	0	0.00	0	0.00	1	100.00
Gastric ulcer	1	1.11	1	0	0	0.00	0	0.00	0	0.00	0	0.00	0	0.00	1	100.00
Flatulence	1	1.11	0	1	0	0.00	0	0.00	0	0.00	0	0.00	1	100.00	0	0.00
Endometrial hemorrhage	1	1.11	1	0	0	0.00	0	0.00	0	0.00	0	0.00	1	100.00	0	0.00
Bacterial infection	1	1.11	1	0	0	0.00	0	0.00	0	0.00	0	0.00	0	0.00	1	100.00
Gastric perforation	1	1.11	1	0	0	0.00	0	0.00	0	0.00	0	0.00	1	100.00	0	0.00
Total	90	100.00	43	49	13	14.44	2	2.22	6	6.67	27	30.00	17	18.89	26	28.89

A Chi-square test performed during correspondence analysis yielded a value of 198.433, suggesting a marked correlation between age-season and morphological diagnosis type and confirming the effectiveness of the corresponding analysis. The cumulative inertia ratio of the first and second dimensions was 0.582, indicating that the first two dimensions explained 58.2% of the total variance. Thus, after dimensionality reduction, the two-dimensional correspondence analysis graph effectively captured the relationship between these variables.

**Figure 1:**
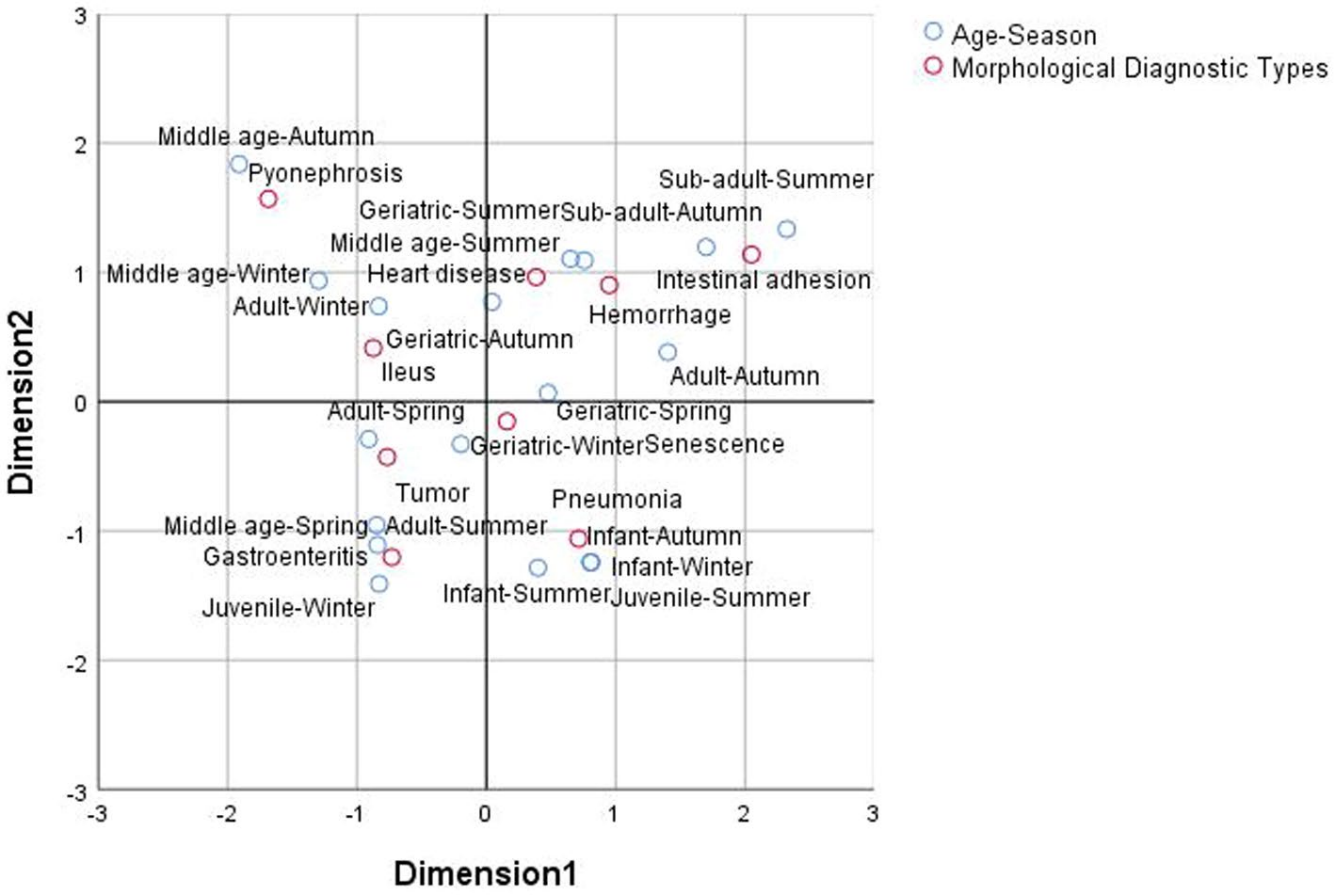
Age-season and morphological diagnosis types chart of correspondence analysis in all François’ langurs.

The correspondence analysis diagram is shown (Figure 1). The X and Y axes intersect at the origin of coordinates (0, 0), dividing the coordinate plane into four quadrants. The positioning of the age-season and morphological diagnosis type variables in the figure enabled approximate categorization into four groups: upper right quadrant, upper left quadrant, lower left quadrant, and lower right quadrant. For interpretation, starting from the origin of the coordinates, points representing column variables (age-season) and points representing row variables (morphological diagnosis type) that are proximate and in the same direction are considered strongly correlated; conversely, points that are distant or not in the same direction are considered weakly correlated (26). In this study, if specific age-season was in the same direction and closed to the morphological diagnosis type suggested a preference of the former for the latter. Subsequent analyses were interpreted and analyzed according to this standard and combined with the data (Table 2).

Pneumonia was located in the lower right quadrant, belonging to the fourth category. Infant-summer, infant-autumn, infant-winter, and juvenile-summer were proximate and aligned in the same direction, indicating that pneumonia-related deaths (*n* = 11) mostly occurred in infants (7, 63.64%) and juveniles (1, 9.09%) (male-to-female ratio of 9:2) and primarily in spring and summer, male individuals have more morbidity. Three cases were diagnosed as lobar pneumonia, with one case also presenting with pulmonary hemorrhage. Catarrhal pneumonia was diagnosed once, with the remaining cases were diagnosed as acute pneumonia, triggered by cold or physical weakness due to changes in the weather. These animals showed increased susceptibility to thermal and cold environmental stress, leading to maladaptation and disease.

Neoplasia is located in the lower left quadrant, falling under the third category. Adult-spring and geriatric-winter variables were proximate and in the same direction, indicating that neoplasia-related deaths (*n* = 7) predominantly occurred in adults (4, 57.14%) and geriatrics (2, 28.57%) (male-to-female ratio of 3:4) and mostly in spring and winter. Three male cases of malignant neoplasia with similar clinical manifestations and development were diagnosed as nasopharyngeal carcinoma. One case of malignant neoplasia was dominated by undifferentiated non-keratinous carcinoma, affecting the nose, eyes, and brain, requiring further examination for precise classification. Other neoplasia types included uterine fibroids, osteosarcoma, and spinal neoplasia.

Ileus was situated in the upper left quadrant, falling under the second category. Adult-winter, adult-spring and middle-age-winter variables were proximate and aligned, indicating that enteropraxis-related deaths (*n* = 7) generally occurred in adult (3, 42.86%) langurs (male-to-female ratio of 4:3) and primarily in spring and winter. These fatalities often resulted from complications related to the consumption of plant food, notably the incomplete digestion of plant fibers, leading to intestinal obstructions, often involving the colon and small intestine. These obstructions then progressed to intestinal torsion, intussusception, inflammation, bleeding, and necrosis, eventually causing systemic infection and death.

Senescence was located in the lower right quadrant, belonging to the fourth category. Geriatric-spring and geriatric-winter variables were closest and aligned, indicating that aging-related death (*n* = 6) only occurred in geriatric langurs (male-to-female ratio of 5:1), primarily in spring and winter. As François’ langurs age, particularly in their middle-age and geriatric stages, various organs, such as the cardiac, lung, liver, and renal, tend to exhibit some degree of damage and lesions, often leading to organ failure.

Gastroenteritis was located in the lower left quadrant, belonging to the third category. Juvenile-winter, middle-age-spring, adult-summer, adult-spring, and geriatric-winter variables were proximate and aligned in same direction, most notably middle-age-spring and adult-summer. The findings indicate that gastroenteritis-related deaths (*n* = 6) were observed across infants to geriatrics, although were more common in adults (2, 33.33%) and middle-aged individuals (2, 33.33%) (male-to-female ratio of 1:1), and predominantly occurred in spring and summer. Most cases were due to bacterial infections leading to severe gastrointestinal inflammation and secondary systemic sepsis; one specific case involved acute *Escherichia coli* hemorrhagic colitis.

Cardiac disease was positioned in the upper right quadrant, belonging to the first category, and most closely aligned with the geriatric-summer variable. This indicated that cardiac-related deaths (*n* = 5) only occurred in male langurs and primarily affected geriatric langurs (3, 60%), with a higher incidence in summer. In addition to sudden cardiovascular fatalities, François’ langurs were also susceptible stress-related deaths due to congenital cardiac disease.

Hemorrhage was found in the upper right quadrant, belonging to the first category. Sub-adult-autumn, adult-autumn, middle-age-summer, and geriatric-summer variables were most proximate and aligned in the same direction, suggesting that hemorrhage-related deaths (*n* = 5) predominantly occurred in geriatric (2, 40%), sub-adult (1, 20%), adult (1, 20%), and middle-aged langurs (1, 20%), with a higher incidence in summer and autumn (male-to-female ratio of 1:4). As social animals, François’ langurs often succumbed fatal outcomes from excessive blood loss due to trauma or internal bleeding during pursuits and social interactions.

Intestinal adhesion was positioned in the upper right quadrant of Figure 1, classified under the first category. Sub-adult-autumn, sub-adult-summer, adult-autumn, middle-age-summer, and geriatric-summer were located in the same direction, with sub-adult-autumn and sub-adult-summer being the closest. These results suggest that intestinal adhesion-related deaths (*n* = 4) were most common in sub-adults (2, 50%), as well as adult and geriatric populations (male-to-female ratio of 3:1), with a higher incidence in autumn. Intestinal adhesion led to a cascade of digestive and respiratory dysfunctions, ultimately resulting in death due to pain and sepsis.

Pyelonephritis resided in the upper left quadrant, classified under the second category. Middle-age-autumn and middle-age-winter were proximate and aligned in the same direction, suggesting that pyelonephritis-related deaths (*n* = 4) were most common in geriatric langurs (3, 75%) and in autumn and winter. These deaths were only found in females.

### 3.3 Systems and organs affected

The frequency of body system and organ lesions causing death (in total numbers and percentages), as well as the count and percentage of males and females and their distributions across age groups is outlined (Table 3). Results showed that the gastrointestinal system was the most affected (*n* = 29, 35.37%), followed by the respiratory system (*n* = 17, 18.89%), multisystem diseases (*n* = 16, 17.78%), cardiovascular system (*n* = 15, 16.67%), urogenital system (*n* = 8, 8.89%), musculoskeletal system (*n* = 3, 3.33%), and special senses (*n* = 2, 2.22%).

**Table 3:** Primary causes of mortality in all François’ langurs by system and organ (undetermined causes not included; *n* = 90).

System/organ	Total		Sex		Infant		Juvenile		Sub-adult		Adult		Middle-age		Geriatric	
	*n*	%	F	M	*n*	%	*n*	%	*n*	%	*n*	%	*n*	%	*n*	%
Gastrointestinal (Total)	29	35.37	12	17	2	6.90	1	3.45	3	10.34	12	41.38	6	20.69	5	17.24
Intestine	19	65.52	7	12	1	5.26	1	5.26	2	10.53	8	42.11	4	21.05	3	15.79
Stomach	5	17.24	2	3	1	20.00	0	N/A	0	N/A	1	20.00	2	40.00	1	20.00
Liver	4	13.79	2	2	0	N/A	0	N/A	1	25.00	2	50.00	0	N/A	1	25.00
Pancreas	1	3.45	1	0	0	N/A	0	N/A	0	N/A	1	100.00	0	N/A	0	N/A
Respiratory (Total)	17	18.89	3	14	7	41.18	1	5.88	1	5.88	1	5.88	0	N/A	3	17.65
Lung	13	76.47	2	11	7	53.85	1	7.69	1	7.69	1	7.69	0	N/A	3	23.08
Nasopharynx	4	23.53	1	3	0	N/A	0	N/A	0	N/A	2	50.00	1	25.00	1	25.00
Multisystem (Total)	16	17.78	10	6	4	25.00	0	N/A	1	6.25	2	12.50	1	6.25	8	50.00
Cardiovascular (Total)	15	18.29	5	10	0	N/A	0	N/A	1	6.67	5	33.33	3	20.00	6	40.00
Head	8	53.33	5	3	0	N/A	0	N/A	1	12.50	3	37.50	1	12.50	3	37.50
Blood	7	46.67	0	7	0	N/A	0	N/A	0	N/A	2	28.57	2	28.57	3	42.86
Urogenital (Total)	8	9.76	8	0	0	N/A	0	N/A	0	N/A	3	37.50	4	50.00	1	12.50
Renal	6	75.00	6	0	0	N/A	0	N/A	0	N/A	2	33.33	3	50.00	1	16.67
Uterus	2	25.00	2	0	0	N/A	0	N/A	0	N/A	1	50.00	1	50.00	0	N/A
Musculoskeletal (Total)	3	3.66	3	0	0	N/A	0	N/A	0	N/A	2	66.67	0	N/A	1	33.33
Bone	2	66.67	2	0	0	N/A	0	N/A	0	N/A	1	50.00	0	N/A	1	50.00
Peritoneal cavity	1	33.33	1	0	0	N/A	0	N/A	0	N/A	1	100.00	0	N/A	0	N/A
Special Senses (Total)	2	2.44	1	1	0	N/A	0	N/A	0	N/A	1	50.00	1	50.00	0	N/A
Eye	2	100.00	1	1	0	N/A	0	N/A	0	N/A	1	50.00	1	50.00	0	N/A

Gastrointestinal system-related mortality accounted for 29 of the 90 deaths (35.37%) and was observed in all age groups, with males outnumbering females (*F* = 12; *M* = 15) and the highest incidence occurring in adults (12, 41.38%).

Intestinal tract lesions accounted for 19 of the 29 gastrointestinal system-related deaths (65.52%), including seven cases of ilues, five cases of gastroenteritis, four cases of intestinal adhesion, one case of intussusception, one case of intestinal perforation, and one case of colitis (hemorrhagic). Gastric diseases accounted for 17.24% (five cases) of gastrointestinal mortality, including gastric ulcer, gastric perforation, gastric dilation, bloat, and gastric relaxation. Hepatic lesions (*n* = 4) accounted for 13.79% of deaths, including three cases of liver abscess and one case of cirrhosis. In addition, one adult female was reported to have died from pancreatitis.

Respiratory system-related mortality accounted for 17 of the 90 deaths (18.89%), with a markedly higher occurrence in males (*F* = 3; *M* = 14) and in infants. Pneumonia was the most common cause of death (11, 64.71%). Of these, three cases were diagnosed as lobar pneumonia, with one case accompanied by pulmonary hemorrhage, edema, and purulent inflammatory lesions. Catarrhal pneumonia was diagnosed once, while the remaining cases were acute pneumonia induced by cold or physical weakness following changes in the weather. Two deaths were attributed to cardiopulmonary failure caused by iatrogenic aspiration pneumonia during treatment. Three male langurs were diagnosed with nasopharyngeal carcinoma and one adult female was diagnosed with malignant neoplasms, characterized by undifferentiated non-keratinous carcinoma of the nose, eyes, and brain, necessitating further investigation for specific neoplasia type determination, with all cases originating in the nasal area.

Multisystem disease-related mortality accounted for 16 of the 90 deaths (17.78%), predominantly observed in geriatric (8, 50.00%) and infant animals (4, 25.00%) and more commonly in females than in males (*F* = 10; *M* = 6). The primary causes of death were degenerative diseases (6, 37.50%), multiple organ abscesses (2, 12.50%), hyperthermia (2, 12.50%), and wasting (2, 12.50%).

Cardiovascular system-related mortality accounted for 15 of the 90 deaths (16.67%), primarily occurring in geriatric (6, 40%), adult (5, 33.33%), and middle-aged langurs (3, 20%), with a male-to-female ratio of 2:1. Deaths resulting from cardiovascular disease were subdivided into vascular system lesions (8, 53.33%) and cardiac-related lesions (7, 46.67%) (male-to-female ratio of 3:5). These deaths included three cases of sepsis, four cases of excessive blood loss due to skin trauma or chest and abdominal injuries sustained in fights, and one case (middle-aged female) of cerebrovascular complications following an accident. Cardiac-related mortality occurred exclusively in males, with three deaths attributed to stress from congenital cardiac disease, two cases recorded as sudden cardiovascular death, and two cases of myocarditis.

Urogenital system-related mortality accounted for eight of the 90 deaths (8.89%), exclusively in females and most commonly in adult (3, 37.5%) and middle-aged animals (4, 50.00%). Renal disease (6, 75.00%) accounted for almost all cases, including renal abscess (4/6, 66.67%) and nephritis (2/6, 33.33%). Two cases of uterine lesions were also recorded, including fibroid lesions and endometrial bleeding.

Musculoskeletal system-related mortality accounted for three of the 90 deaths (3.33%), all occurring in female langurs. This included a geriatric langur with osteosarcoma, an adult with a benign spinal neoplasia, and another case involving acute herniosis in the abdomen.

Deaths attributed to specific sensory lesions, which accounted for two of the 90 deaths (2.22%), were caused by acute conjunctivitis resulting from infections and inflammation in the eye, triggered by injuries sustained during fights.

### 3.4 Etiological agents

The etiologies leading to death (in total numbers and percentages), as well as the count and percentage of males and females, and their distributions across age groups for each etiology were outlined (Table 4). The most common etiologies were infectious or inflammatory (*n* = 32, 35.56%), physiological (*n* = 17, 18.89%), bacterial (*n* = 7, 7.78%), neoplastic (*n* = 7, 7.78%), degenerative (*n* = 7, 7.78%), traumatic (*n* = 5, 5.56%), idiopathic (*n* = 5, 5.56%), parasitic (*n* = 3, 3.33%).

**Table 4:** Primary causes of mortality in all François’ langurs by etiology (undetermined causes not included) (*n* = 90).

Etiology	Total		F	M	Infant	Juvenile	Sub-adult	Adult	Middle-age	Geriatric
	*n*	%	*n*	*n*	*n*	*n*	*n*	*n*	*n*	*n*
Infectious/Inflammatory	32	35.56	16	16	8	2	2	9	6	5
Physiological	17	18.89	5	12	1	0	2	7	4	3
Bacterial	7	7.78	3	4	0	0	0	4	1	2
Neoplastic	7	7.78	4	3	0	0	0	4	1	2
Degenerative	7	7.78	5	2	0	0	0	0	0	7
Traumatic	5	5.56	4	1	0	0	1	1	1	2
Idiopathic	5	5.56	0	5	0	0	0	1	1	3
Parasitic	3	3.33	1	2	0	0	0	2	1	0
Iatrogenic	2	2.22	0	2	0	0	1	0	0	1
Environmental	2	2.22	1	1	2	0	0	0	0	0
Nutritional	2	2.22	2	0	2	0	0	0	0	0
Other	1	1.11	1	0	0	0	0	0	1	0
Total	90	100.00	42	48	13	2	6	28	16	25

Infections and inflammation (*n* = 32) were most common in adult (9, 28.13%), juvenile (8, 25.00%), middle-aged (6, 18.75%), and geriatric langurs (5, 15.63%), with an equal ratio of males and females (1:1). The most affected systems were the gastrointestinal (11, 34.38%), respiratory (11, 34.38%), and genitourinary systems (6, 18.75%). The etiology of infection was frequently correlated with diseases such as enteritis, pneumonia, renal abscess, and hepatic abscess.

Mortality attributed to physiological causes (*n* = 17) predominantly occurred in adult (7, 41.18%), middle-aged (4, 23.53%), and geriatric François’ langurs (3, 17.65%), with a male-to-female ratio of 12:5. These deaths, associated exclusively with the gastrointestinal system, frequently involved ileus (7, 41.18%) and intestinal adhesions (4, 23.53%).

Bacterial-related mortality (*n* = 7) was most common in adults (4, 57.14%) and geriatric langurs (2, 28.57%), with a male-to-female ratio of 4:3. The cardiovascular system was the most affected, with bacterial infection-induced septicemia and myocarditis being the primary lesions observed.

Neoplastic-related mortality (*n* = 7) was generally seen in adult (4, 57.14%) and geriatric langurs (2, 28.57%), with a male-to-female ratio of 3:4. The respiratory and musculoskeletal systems were the most affected, with prevalent diseases being malignant neoplasia such as undifferentiated non-keratinized carcinoma of the nose, eyes, and brain, uterine fibroids, osteosarcoma, and spinal neoplasia.

Degenerative-related mortality (*n* = 7) only occurred in geriatric François’ langurs, with a male-to-female ratio of 2:5. The multisystem was the most impacted, with senescence and cirrhosis as common pathologies.

Mortality due to trauma (*n* = 5) occurred in sub-adult to geriatric langurs, with a male-to-female ratio of 1:4. Hemorrhage, affecting only the cardiovascular system, was the most common morphological diagnosis linked to trauma.

Mortality from idiopathic etiology (*n* = 5) occurred exclusively in male François’ langurs, most commonly in geriatric animals (3, 60%). Only the cardiovascular system was implicated in these cases.

### 3.5 Mortality by age group

Mortality resulting from various morphological diagnoses, systems, and etiologies varied across age groups and was tabulated as presents the top five diagnoses for each of the six age groups (Table 5). The percentage of mortalities in each age group categorized by system (Table 6) and the percentage of mortalities in each age group by confirmed etiology (Table 7).

**Table 5:** The five most common morphological diagnoses by age group (undetermined causes not included; *n* = 90).

Infant			Juvenile			Sub-adult		
Morphological diagnosis	*n*	%	Morphological diagnosis	*n*	%	Morphological diagnosis	*n*	%
Pneumonia	7	53.85	Pneumonia	1	50.00	Intestinal adhesion	2	33.33
Hyperthermia	2	15.38	Gastroenteritis	1	50.00	Hepatapostema	1	16.67
Inanition	2	15.38				Foreign body asphyxia	1	16.67
Gastroatonia	1	7.69				Hemorrhage	1	16.67
Gastroenteritis	1	7.69				Multiple organ abscesses	1	16.67
Total	13	100.00	Total	2	100.00	Total	6	100.00
Adult			Middle-age			Geriatric		
Morphological diagnosis	*n*	%	Morphological diagnosis	*n*	%	Morphological diagnosis	*n*	%
Neoplasia	4	33.33	Pyonephrosis	3	37.50	Senescence	6	40.00
Enteropraxis	2	16.67	Gastroenteritis	2	25.00	Cardiac disease	3	20.00
Gastroenteritis	2	16.67	Myocarditis	1	12.50	Gastroenteritis	2	13.33
Septicemia	2	16.67	Intestinal obstruction	1	12.50	Neoplasia	2	13.33
Hepatapostema	2	16.67	Gastric perforation	1	12.50	Hemorrhage	2	13.33
Total	12	100.00	Total	8	100.00	Total	15	100.00

**Table 6:** Percentage of mortalities in each age group by system in all François’ langurs (undetermined causes not included; *n* = 90).

System	Infant		Juvenile		Sub-adult		Adult		Middle-age		Geriatric	
	*n*	%	*n*	%	*n*	%	*n*	%	*n*	%	*n*	%
Gastrointestinal system	2	15.38	1	50.00	3	50.00	12	42.86	6	37.50	5	20.00
Cardiovascular system	0	N/A	0	N/A	1	16.67	5	17.86	3	18.75	6	24.00
Respiratory system	7	53.85	1	50.00	1	16.67	3	10.71	1	6.25	4	16.00
Genitourinary system	0	N/A	0	N/A	0	N/A	3	10.71	4	25.00	1	4.00
Multisystem	4	30.77	0	N/A	1	16.67	2	7.14	1	6.25	8	32.00
Musculoskeletal system	0	N/A	0	N/A	0	N/A	2	7.14	0	N/A	1	4.00
Special sense	0	N/A	0	N/A	0	N/A	1	3.57	1	6.25	0	N/A
Total	13	100.00	2	100.00	6	100.00	28	100.00	16	100.00	25	100.00

**Table 7:** Percentage of mortalities in each age group by etiology in all François’ langurs (undetermined causes not included; *n* = 90).

Etiology	Infant		Juvenile		Sub-adult		Adult		Middle-age		Geriatric	
	*n*	%	*n*	%	*n*	%	*n*	%	*n*	%	*n*	%
Infectious/Inflammatory	8	61.54	2	100.00	2	33.33	9	32.14	6	37.50	5	20.00
Physiological	1	7.69	0	N/A	2	33.33	7	25.00	4	25.00	3	12.00
Bacterial	0	N/A	0	N/A	0	N/A	4	14.29	1	6.25	2	8.00
Neoplastic	0	N/A	0	N/A	0	N/A	4	14.29	1	6.25	2	8.00
Degenerative	0	N/A	0	N/A	0	N/A	0	N/A	0	N/A	7	28.00
Traumatic	0	N/A	0	N/A	1	16.67	1	3.57	1	6.25	2	8.00
Idiopathic	0	N/A	0	N/A	0	N/A	1	3.57	1	6.25	3	12.00
Parasitic	0	N/A	0	N/A	0	N/A	2	7.14	1	6.25	0	N/A
Iatrogenic	0	N/A	0	N/A	1	16.67	0	N/A	0	N/A	1	4.00
Environmental	2	15.38	0	N/A	0	N/A	0	N/A	0	N/A	0	N/A
Nutritional	2	15.38	0	N/A	0	N/A	0	N/A	0	N/A	0	N/A
Other	0	N/A	0	N/A	0	N/A	0	N/A	1	6.25	0	N/A
Total	13	100.00	2	100.00	6	100.00	28	100.00	16	100.00	25	100.00

Infant mortality (*n* = 13) was predominantly due to pneumonia (7, 53.85%), hyperthermia (2, 15.38%), and inanition (2, 15.38%). Respiratory systems (7, 53.85%) and multisystem diseases (4, 30.77%) were the most affected. Infants with established etiologies typically died from infectious (8, 61.54%), environmental (2, 15.38%), and nutritional (2, 15.38%) causes.

Juvenile mortality (*n* = 2) was primarily due to pneumonia (1, 50.00%) and gastroenteritis (1, 50.00%), involving the respiratory and gastrointestinal systems, with death from infectious causes.

Sub-adult mortality (*n* = 6) was mainly from intestinal adhesion (2, 33.33%), hepatapostema (1, 16.67%), foreign body asphyxia (1, 16.67%), hemorrhage (1, 16.67%), and multiple organ abscesses (1, 16.67%). The gastrointestinal system was most often involved, and death was usually due to infectious (2, 33.33%) and physiological causes (2, 33.33%).

Adult mortality (*n* = 28) resulted from more varied causes, including neoplasia (4, 33.33%) intestinal obstruction (2, 16.67%), gastroenteritis (2, 16.67%), septicemia (2, 16.67%), and hepatic abscess (2, 16.67%). The most affected systems were the gastrointestinal (12, 42.86%), cardiovascular (5, 17.86%), respiratory (3, 10.71%), and genitourinary systems (3, 10.71%). Adults with established etiologies typically died from infectious/inflammatory (9, 32.14%), physiological (7, 25.00%), bacterial (4, 14.29%), and neoplastic (4, 14.29%) causes.

Middle-age mortality (*n* = 16) primarily resulted from renal abscess (3, 37.50%), gastroenteritis (2, 25.00%), myocarditis (1, 12.50%), intestinal obstruction (1, 12.50%) and gastric perforation (1, 12.50%). The most affected systems were the gastrointestinal (6, 37.50%), genitourinary (4, 25.00%), and cardiovascular systems (3, 18.75%). Middle-aged langurs with established etiologies usually died from infectious or inflammatory (6, 37.50%) and physiological (4, 25.00%) causes.

Geriatric mortality (*n* = 25) was predominantly the result of senescence (6, 40.00%), cardiac disease (3, 20.00%), intestinal obstruction (2, 13.33%), neoplasia (2, 13.33%), and hemorrhage (2, 13.33%). The most affected systems were multisystem disease (8/25, 32.00%), cardiovascular system (6/25, 24.00%), gastrointestinal system (5/25, 20.00%), and respiratory system (4/25, 25.00%). Geriatric langurs with established etiologies usually died from degenerative (7, 28.00%), infectious (5, 20.00%), physiological (3, 12.00%), and idiopathic (3, 12.00%) causes.

## 4 Discussion

This study entailed a detailed examination of mortality among François’ langurs, encompassing morphological diagnosis, organ system, and etiological analysis of 97 individuals over a 16-year period (2007–2022). The predominant morphological diagnoses, accounting for 58.89% of all deaths, included pneumonia, neoplasia, senescence, gastroenteritis, intestinal obstruction, cardiac disease, hemorrhage, intestinal adhesion, and renal abscess. Gastrointestinal system was the most frequently affected, followed by respiratory system, multisystem diseases, cardiovascular system, genitourinary system, musculoskeletal system, and sensory organs. The leading causes of death, in descending order, were infectious/inflammatory, physiological, bacterial, neoplastic, degenerative traumatic, idiopathic, and parasitic diseases, with iatrogenic, environmental, and nutritional causes accounting for less than 3% each.

### 4.1 Main morphological diagnosis of François’ langurs

Pneumonia emerged as the leading morphological diagnosis in this study, accounting for the highest proportion of cases (11/90, 12.22%) and most frequently affecting infants and juveniles. The prevalence of pneumonia is closely connected to pathogenic microorganisms, environmental conditions, and the physical health of animals. In managed settings, the François’ langurs are at a heightened risk of encountering pathogens, thereby increasing their vulnerability to diseases. Naturally residing in tropical and subtropical areas, these primates are particularly sensitive to abrupt temperature declines. Rapid temperature fluctuations in managed care can often result in respiratory infections and pneumonia. Infants and juveniles, with their comparatively weaker physical constitutions and less developed immune systems, are more prone to pathogenic invasion, which can result in lung infections and pneumonia. Thus, it is crucial that primate enclosures remain dry and warm, especially during seasonal changes. Vigilant monitoring of changes in appetite and behavior is also essential to ensure timely intervention when necessary.

The results highlighted a notable mortality rate due to malignant neoplasia (7/90, 7.78%). Three cases were identified as nasopharyngeal carcinomas with similar clinical signs throughout, while another was diagnosed as an undifferentiated non-keratinous carcinoma, potentially causing damage to the brain, eyes, and nose. The pathogenesis of nasopharyngeal carcinoma typically involves genetic, viral, and environmental factors and it is particularly prevalent in southern China (26). Malignant neoplasia are frequently observed in monkey populations and have been reported to result in high mortality rates in François’ langurs, including cases of oral and nasal squamous cell carcinoma (13), lymphosarcoma (11), and pyloric adenocarcinoma (16). Treating cancer, typically managed in humans with radiotherapy, chemotherapy, or surgery, poses significant challenges in François’ langurs. Limitations in equipment availability and the complexity of intraoperative and postoperative care make these methods impractical, leading to the adoption of more conservative treatments akin to traditional Chinese medicine, as applied in species such as François’ langurs. Nevertheless, neoplasia interventions often fail to provide protection, emphasizing the importance of preventative measures such as enhanced feeding, management, and early health examinations. Currently, however, the institute faces shortages in personnel, technology, and equipment. Thus, to elevate standards of monitoring and disease prevention, it is crucial to secure financing and technical support through various channels.

### 4.2 Affected systems

In managed care, gastrointestinal disorders are a significant factor in the high mortality rates of François’ langurs. Pereira et al. (27) conducted a 19-year retrospective study of postmortem morbidity and mortality in managed care langurs (*Trachypithecus* spp.) (*n* = 88) at six zoological organizations in the United Kingdom, revealing gastrointestinal diseases and systemic infections as primary causes of death. Similarly, Nong et al. (28) analyzed 615 managed care François’ langurs at Nanning Zoo in China and found that gastroenteritis and food stagnation-induced gastrodilation were prevalent causes of death, with the digestive, respiratory, and urogenital systems being the most affected. These findings are consistent with the current, showing the highest mortality rates fromgastrointestinal system diseases, especially gastrointestinal diseases such as gastroenteritis, intestinal obstruction, gastrointestinal relaxation, gastric dilatation, gastric perforation, gastric ulcer, and intussusception. The high mortality rate from digestive diseases in François’ langurs is linked to the complex structure of their stomachs. As folivorous primates, they possess a complex stomach, including features such as cystic fundus, gastric tubes, enlarged cecum and colon, and enlarged stomach surface area (29, 30). Their digestive physiology is adapted for cellulose decomposition by stomach-residing bacteria, efficiently digesting and fermenting leaf fiber. However, their ability to digest carbohydrate-rich and protein feeds is limited. Various health ailments that affect François’ langurs are attributed to environmental shifts and artificial feeding and care challenges. Their managed diet, often high in soluble sugars and low in fiber, differs significantly from their natural diet adapted to crude fiber digestion. This dietary shift, to feeds high in starch and sugar, can disrupt their digestive function and lead to gastrointestinal diseases. The limited space in managed care and subsequent reduction in activity, coupled with a timid nature and sensitivity to environmental stress, may also lead to an increased incidence of gastrointestinal diseases in François’ langurs. Other contributing factors to gastrointestinal issues include insufficient drinking water, ingestion of foreign bodies, poor-quality feed, improper feeding management, and intestinal parasitic infections. For instance, intestinal obstructions from trichobezoars are common in long-term managed-contained monkeys (5), with severe fibrinopurulent and proliferative peritonitis due to indigestible food also reported in northern plains gray langurs (*Presbytis entellus*) (31). Thus, gastrointestinal diseases are a prevalent issue in managed primates, necessitating urgent attention. Yang et al. (32) successfully used traditional Chinese medicine to treat intestinal obstruction in François’ langurs. Research has also shown that crude fiber content significantly influences food selection in langurs (33). As such, it is advocated for the establishment of a carefully managed, species-appropriate feeding regime for managed care François’ langurs, with feed formula optimization based on their natural habits and digestive functionality. Additionally, it is recommended diet customization based on the langurs’ appetite and adjusting the balance of cake to green feed in their diet to align with seasonal variations and age-related differences. Furthermore, traditional Chinese medicine can be employed for dietary conditioning. The diet for François’ langurs should primarily consist of high-fiber green roughage, such as fresh branches, leaves, flowers and fruits, supplemented by grass, green leafy vegetables, and concentrated feed. This diet aims to reduce soluble sugar intake and increase dietary fiber intake, avoiding high-fat feeds. The feed should contain adequate crude protein, carbohydrates, crude fiber, trace elements, and various vitamins, with frequent small meals to mimic wild feeding patterns. Additionally, minimizing artificial stress is crucial for reducing the incidence of secondary gastrointestinal diseases.

It is public knowledge that cardiovascular diseases are a complex and common condition in managed care primates. Our current review found that the cardiac abnormalities of François’ langurs were mainly manifested as cardiomegaly with hemorrhagic spots, which was associated significantly with male gender. However, in an earlier study published by Flanders et al., they retrospective analysis of adult-onset cardiac disease in François’ langurs housed. It appears that François’ langurs appear to be predisposed to cardiac disease and subsequent death when compared with similar langur species. Older animals and male animals are predisposed to develop cardiac fibrosis (34). Recent reports of idiopathic and infectious diseases along with disorders of the cardiovascular, respiratory, and gastrointestinal body systems were particularly prominent in the nonhuman primate literature. Therefore, we recommended that the relevant staff regularly carry out disease screening tests on langurs, to diagnose affected animals as early as possible, to monitor progression of disease, and to guide treatment.

### 4.3 Mortality rates in different age groups

Acute pneumonia and malnutrition due to colds was prevalent illnesses among infant François’ langurs. Malnutrition in these infants is frequently caused by insufficient milk production by their mothers, leading to abandonment (35), insufficient nutrition, and consequent excessive thinness and weakness, culminating in their death. Furthermore, malnutrition can reduce their disease resistance, thereby increasing their susceptibility to secondary infections like pneumonia. Respiratory diseases in these young langurs are mainly related to sudden changes in temperature. Given the relatively weaker immunity of François’ langurs during infancy, it is crucial that breeding and management personnel be particularly vigilant and implement appropriate warming measures in response to temperature fluctuations.

The primary cause of death in adult and middle-age François’ langurs was renal disease, neoplasia, gastroenteritis, and intestinal obstruction. Factors such as mycotoxins, plant toxins, and excessive protein intake can harm the renals, with long-term inappropriate feeding leading to chronic renal damage. Neoplasia development is linked to various factors, such as animal age, physical health, living environment, feed mold, and incorrect drug use. Moreover, renal disease is also associated with gastrointestinal microecology; thus, ensuring dietary diversity to meet nutritional requirements while minimizing toxin accumulation from a single-source diet is important (36–38). Consequently, in daily care, optimizing the diet by providing a variety of leaves, reducing high-sugar food, and reducing protein intake can help reduce the occurrence of renal disease in François’ langurs.

Senescence and cardiac disease was the predominant causes of death in older langurs, especially females, spanning a broad age range. As langurs age, the functionality of various bodily systems declines, leading to increased accidents and incidents of sudden death. With an aging langur population, a key focus is on medical management. Therefore, caretakers must enhance feeding practices, conduct thorough health care, and provide nutritional supplements and Chinese herbal preparations to boost the immunity and overall health of elderly langurs, thereby delaying the aging process (19). Particular attention should be given to older female langurs, who often have lower social status within the group and are prone to being chased or bitten by other males or females. To prevent injuries or fatalities resulting from such stress, it is advisable to house elderly langurs in separate, smaller groups (19).

Understanding the causes of mortality in François’ langurs is crucial for shaping future care and management approaches, not only to extend their lifespan but also to enhance their overall welfare. Nevertheless, the current breeding and management practices for François’ langurs, coupled with the constraints of archival records and specific climatic conditions of Wuzhou, indicate that a more thorough investigation and research into the causes of death in artificially bred François’ langurs is necessary.

## Data Availability

The original contributions presented in the study are included in the article/supplementary material, further inquiries can be directed to the corresponding authors.
